# msBayes: Pipeline for testing comparative phylogeographic histories using hierarchical approximate Bayesian computation

**DOI:** 10.1186/1471-2105-8-268

**Published:** 2007-07-26

**Authors:** Michael J Hickerson, Eli Stahl, Naoki Takebayashi

**Affiliations:** 1 Biology Department, Queens College, CUNY, 65-30 Kissena Blvd, Flushing, NY 11367-1597, USA; 2 Department of Biology, University of Massachusetts Dartmouth, 285 Old Westport Rd, North Dartmouth, MA 02747, USA; 3 Institute of Arctic Biology and Department of Biology and Wildlife, 311 Irving I Bldg, University of Alaska, Fairbanks, AK 99775, USA

## Abstract

**Background:**

Although testing for simultaneous divergence (vicariance) across different population-pairs that span the same barrier to gene flow is of central importance to evolutionary biology, researchers often equate the gene tree and population/species tree thereby ignoring stochastic coalescent variance in their conclusions of temporal incongruence. In contrast to other available phylogeographic software packages, msBayes is the only one that analyses data from multiple species/population pairs under a hierarchical model.

**Results:**

msBayes employs approximate Bayesian computation (ABC) under a hierarchical coalescent model to test for simultaneous divergence (TSD) in multiple co-distributed population-pairs. Simultaneous isolation is tested by estimating three hyper-parameters that characterize the degree of variability in divergence times across co-distributed population pairs while allowing for variation in various within population-pair demographic parameters (sub-parameters) that can affect the coalescent. msBayes is a software package consisting of several C and R programs that are run with a Perl "front-end".

**Conclusion:**

The method reasonably distinguishes simultaneous isolation from temporal incongruence in the divergence of co-distributed population pairs, even with sparse sampling of individuals. Because the estimate step is decoupled from the simulation step, one can rapidly evaluate different ABC acceptance/rejection conditions and the choice of summary statistics. Given the complex and idiosyncratic nature of testing multi-species biogeographic hypotheses, we envision msBayes as a powerful and flexible tool for tackling a wide array of difficult research questions that use population genetic data from multiple co-distributed species. The msBayes pipeline is available for download at  under an open source license (GNU Public License). The msBayes pipeline is comprised of several C and R programs that are run with a Perl "front-end" and runs on Linux, Mac OS-X, and most POSIX systems. Although the current implementation is for a single locus per species-pair, future implementations will allow analysis of multi-loci data per species pair.

## Background

Testing for simultaneous divergence (vicariance) across different population-pairs that span the same historical barrier to gene flow is of central importance to evolutionary biology, biogeography and community ecology [1-4]. Such inferences inform processes underlying speciation, community composition, range delineation, and the ecological consequences of climatic changes. Estimating a population divergence time with an appropriate statistical model [5] can be accomplished in a variety of ways [6-8], yet analyzing comparative phylogeographic data with multiple co-occurring species pairs that vary with respect to demographic parameters and pairwise coalescent times is less straightforward.

Instead of conducting an independent analysis on every population-pair and testing the hypothesis of temporal concordance based on this set of independent parameter estimates of divergence time, the hierarchical model employed by msBayes follows the suggestion of [9] by concurrently estimating three hyper-parameters that characterize the mean, variability and number of different divergence events across a set of population-pairs. The model employed in msBayes allows estimation of these hyper-parameters across a multi-species data set while explicitly incorporating uncertainty and variation in the sub-parameters that independently describe each population-pair's demographic history (divergence time, current, ancestral and founding effective population sizes), post-divergence migration rate and recombination rate. The msBayes software pipeline is based on the introduction of the approximate Bayesian computation (ABC) method for sampling from the hyper-posterior distribution for testing for simultaneous divergence [10]. We review the important features here. Although the current implementation is for a single locus per species-pair, future implementations will allow analysis of multi-loci data per species/population pair.

In contrast to previous ABC-like models [11-15], our TSD is accomplished by implementing a hierarchical Bayesian model in which the sub-parameters (Φ; within population-pair parameters) are conditional on "hyper-parameters" (*ϕ*) that describe the variability of Φ among the *Y *population-pairs. For example, divergence times (Φ) can vary across a set of population pairs conditional on the set of hyper-parameters (*ϕ*) that varies according to their hyper-prior distribution. Instead of explicitly calculating the likelihood expression P(Data | *ϕ*,Φ) to get a posterior distribution, we sample from the posterior distribution P((*ϕ*,Φ) | Data) by simulating the data *K *times under the coalescent model using candidate parameters drawn from the prior distribution P(*ϕ*,Φ). A summary statistic vector **D **for each simulated dataset is then compared to the observed summary statistic vector in order to generate random observations from the joint posterior distribution *f*(*ϕ*_*i*_,Φ_*i*_|**D**_*i*_) by way of a rejection/acceptance algorithm [16] followed by an optional weighted local regression step [15]. Loosely speaking, hyper-parameter values are accepted and used to construct the posterior distribution with probabilities proportional to the similarity between the summary statistic vector from the observed data and the summary statistic vector calculated from simulated data.

**Figure 1:**
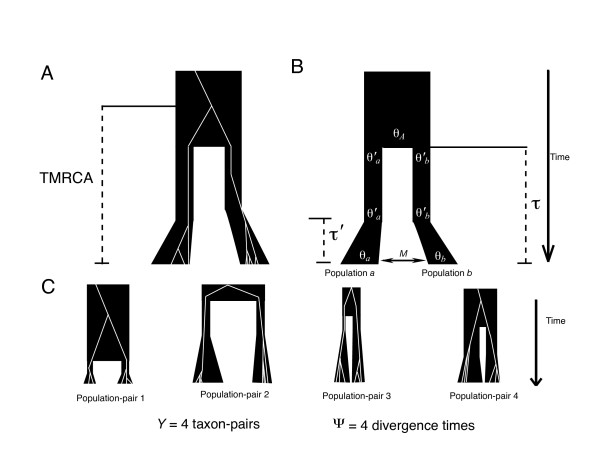
**Depiction of the multiple population-pair divergence model used for the ABC estimates of Ψ, E(*τ*), and Ω**. (A): The white lines depict a gene tree with TMRCA being the time to the gene sample's most recent common ancestor, and the black tree containing the gene tree is the population/species tree. (B): Parameters in the multiple population-pair divergence model. The population mutation parameter, *θ*, is 2*Nμ *where 2*N *is the summed haploid effective female population size of each pair of daughter populations (μ is the per gene per generation mutation rate). The time since isolation of each population pair is denoted by *τ *(in units of 2*N*_*AVE *_generations, where *N*_*AVE *_is the parametric expectation of *N *across *Y *population pairs given the prior distribution). Population mutation parameters for daughter populations *a *and *b *are *θ*_*a *_and *θ*_*b*_, whereas *θ *'_*a *_and *θ'*_*b *_are the population mutation parameters for the sizes of daughter populations *a *and *b *at the time of divergence until *τ' *(length of bottleneck). The daughter populations *θ *'_*a *_and *θ'*_*b *_then grow exponentially to sizes *θ*_*a *_and *θ*_*b*_. The population mutation parameter for each ancestral population is depicted as *θ*_*A*_. The migration rate between each pair of daughter populations is depicted as *M *(number of effective migrants per generation). (C): Example of four population-pairs where parameters in (B) are drawn from uniform priors.

The hierarchical model consists of ancestral populations that split at divergence times *T*_*Y *_= {*τ*_1_...*τ*_*Y*_} in the past. The hyper-parameter set, *ϕ *quantifies the degree of variability in these *Y *divergence times across the *Y *ancestral populations and their *Y *descendent population pairs: (1) Ψ, the number of possible divergence times (1 ≤ Ψ ≤ *Y*); (2) E(*τ*), the mean divergence time; and (3) Ω, the ratio of variance to the mean in these *Y *divergence times, Var(*τ*)/E(*τ*). The sub-parameters for the *i*-th population-pair (Φ_*i*_) are allowed to vary independently across *Y *population pairs and include divergence time (*τ*_*i*_), current population sizes, ancestral population sizes, post-divergence founding population sizes, durations of post-divergence population growth, recombination rates, and post-divergence migration rates. The multiple population-pair splitting model is depicted in Figure 1. Each divergence time parameter *τ *is scaled by 2*N*_*AVE *_generations, where *N*_*AVE *_is the parametric expectation of *N *(effective population size) across *Y *population pairs given the prior distribution.

The summary statistic vector **D **employed in msBayes currently consists of up to six summary statistics collected from each of the *Y *population pairs (*π*,*θ*_*W*_, Var(*π *- *θ*_*W*_), *π*_*net*_, *π*_*b*_, and *π*_*w*_). This includes *π*, the average number of pairwise differences among all sequences within each population pair, *θ*_*W *_the number of segregating sites within each population pair normalized for sample size, [17], Var(*π *- *θ*_*W*_) in each population pair, and *π*_*net*_, Nei and Li's net nucleotide divergence between each pair of populations [18]. This last summary statistic is the difference (*π*_*b *_- *π*_*w*_) where *π*_*b *_is the average pairwise differences between each population pair and *π*_*w *_is the average pairwise differences within a sister pair of descendent populations. The default setting includes the first four aforementioned summary statistics because they were found to be a least correlated subset of a larger group [19], however, future versions of msBayes will allow users to choose other summary statistics.

An extensive simulation study was conducted in [10] to evaluate the performance of our hierarchical ABC model. Because comparative phylogeographic studies are often conducted on multi-species data sets that include rare taxa from which it is difficult to obtain samples from many individuals, we extend the previous evaluation to explore the effectiveness of msBayes in conducting a TSD given small sample sizes (≤ 5 individuals per population pair).

## Implementation

**Figure 2:**
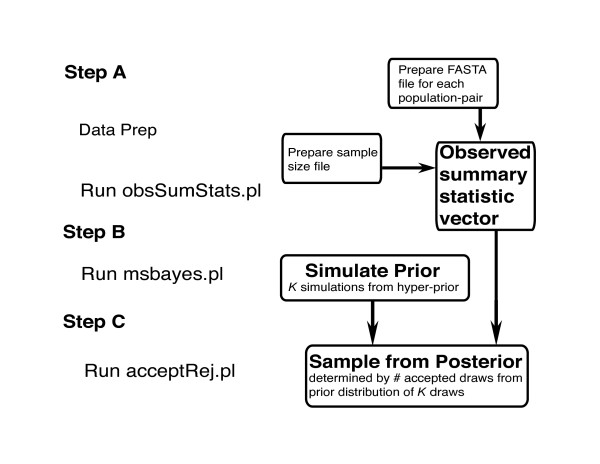
Flowchart describing operation of msBayes.

After preparation of a sample size file and the input files from DNA sequence data, running msBayes is a three step process that includes: (**A**) calculating the observed summary statistic vector from the DNA sequence input files and the sample size file; (**B**) running coalescent simulations of the DNA sequence data using parameters drawn from the hyper-prior (*ϕ*) and prior (Φ); and (**C**) sampling from the posterior distribution and obtaining posterior estimates of Ψ, E(*τ*), and Ω across the *Y *population pairs (Figure 2).

**Step A **is accomplished by a command-line Perl program (obsSumStats.pl) which uses two C programs to calculate the observed summary statistic vector file. Specifically, the user runs obsSumStats.pl after collecting separate aligned DNA sequence files from each population-pair in FASTA format, and constructing an additional text file that describes the samples sizes, length of genes and transition/transversion rate ratios.

**Step B **iteratively simulates data sets under the hierarchical model by: **1.) **randomly drawing hyper-parameters and sub-parameters from the hyper-prior and sub-prior distributions; **2.) **using these hyper-parameters and sub-parameters to simulate finite sites DNA sequence data from *Y *population-pairs; and **3.) **calculating a summary statistic vector **D **from the simulated data set of *Y *population-pairs. This is accomplished with several C programs that are run with a Perl "front-end" (msbayes.pl) that either prompts the user for the upper-bounds of various priors and the number of iterations to simulate or alternatively uses a batch configuration file with equivalent information. The first C program draws hyper-parameters and sub-parameters from their hyper-prior and sub-prior distributions. These parameters are then fed into several C programs that simulate finite-sites DNA sequence data using msarbpopQH a modified version of Hudson's classic coalescent simulator ms [20], which incorporates finite sites mutation and arbitrary population structure and dynamics. Another set of C programs calculates a summary statistic vector (**D**) for every simulated data set of *Y *population pairs.

**Step C **is accomplished by our command-line user-interface program (acceptRej.pl). This Perl program internally uses R for the calculation. The algorithim is a simple extension of the original R scripts which are kindly provided by M. Beaumont [15]. This step does the rejection/acceptance sampling and local regression to produce the approximate sample of the posterior distribution. This third step uses the output of **step B **as the input and produces an output file that contains multiple graphical depictions of the posterior distributions and a text output file with various summaries of the posterior distributions (estimates of Ψ, E(*τ*), and Ω across the *Y *population pairs). The user can choose which summary statistics to include within **D **(the summary statistic vector), choose the proportion of accepted draws from the prior, and can optionally choose to perform simple rejection sampling without the additional local regression step.

We distribute msBayes as C source code and pre-compiled binaries that run on Linux or Mac OS X operating systems. The msBayes package also includes the R functions, and Perl scripts, as well as installation/running instructions.

## Results and Discussion

### Performance of estimator with small sample sizes

At the present time, there are no other available coalescent-based tools for analyzing multiple population pairs simultaneously to yield hyper-parameter estimates. Although IM and IMa are most similar to msBayes [8,21] because they estimate divergence times and population sizes from single pairs of populations under a coalescent model, these do not employ a hierarchical model and therefore can only do so one pair at a time. The program MCMCcoal can estimate divergence times of a known phylogeny under a coalescent model, but can only use the separate divergence time estimates to test for phylogeographic congruence [7]. The program BEST [6] infers a species phylogeny and demographic parameters (e.g. divergence times and population sizes) using a population coalescent model, but likewise can only use the individual divergence time estimates to test for phylogeographic congruence across a multi-species dataset. On the other hand, the hierarchical model employed in msBayes not only can estimate hyper-parameters but also comes with the benefit of additional information gained from the "borrowing strength" across datasets [22-24]. In this case, the resulting emergent multi-species hyper-estimates use more of the information than the sum of their parts (within species-pair estimates).

Although the hierarchical ABC model employed in msBayes was extensively evaluated in [10], the behavior of the ABC estimator given minimal sampling of individuals was not examined. Because comparative phylogeographic studies are often conducted on multi-species data sets that include rare taxa from which it is difficult to obtain samples from many individuals, we evaluate how low sample sizes can affect inference. To this end, we explored the performance in scenarios where ≤ 5 per population pair were sampled from each of 10 population pairs. We created 1,000 simulated data sets under each of two different histories: (1) simultaneous divergence history and (2) variable divergence history among population pairs. In the simultaneous divergence history (true Ω = Var(*τ*)/E(*τ*) = 0), all ten population pairs arose from ancestral populations at *τ *= 1.8 before the present. In the variable divergence history (true Ω = 0.1), two population pairs arose at *τ *= 1.0 and eight population pairs arose at *τ *= 2.0 before the present. We simulated these two histories with small sample sizes (2–5 individual per population-pair) and with larger sample sizes (20 individuals per population pair; 10 per descendent population). The simulated data sets consisted of haploid mtDNA samples from ten population pairs that were 550–600 base pairs in length. From each of the four sets of 1,000 simulated data sets, we used msBayes to obtain 1,000 ABC estimates of the hyper-parameter, Ω, with the goal of assessing the effects of sample sizes on the root mean square error (RMSE) of the ABC Ω estimator. Each estimate of Ω was obtained from the mode of 1,000 accepted draws (after the local regression step) from 500,000 random draws from the hyper-prior, as these conditions were found to be optimal in [10]. For the larger sample sizes we use four classes of summary statistics (*π*, *θ*_*W*_, Var(*π *- *θ*_*W*_) and *π*_*net*_), while for the smaller sample sizes we only use *π*_*b *_to avoid null or n.a.n. values (not a number) that are yielded when only one sample is collected from a descendent population.

**Figure 3:**
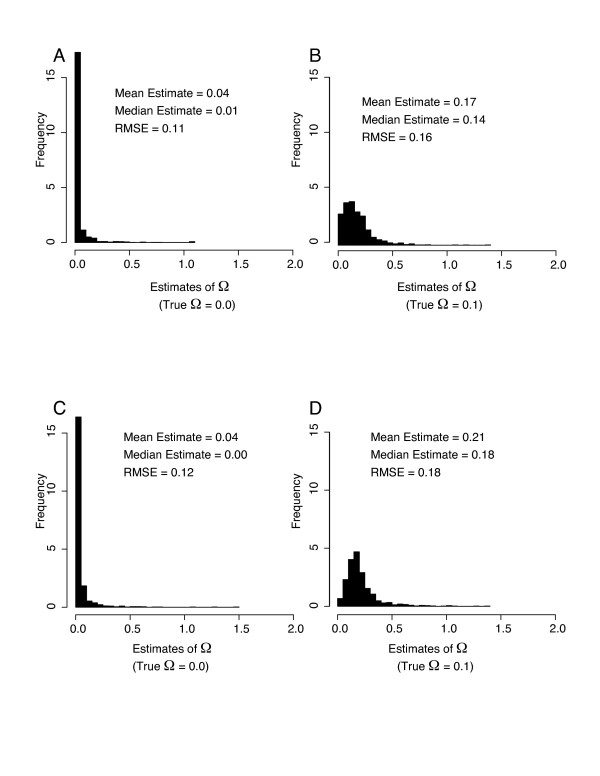
Performance of estimator. Panels A through D each depict frequency histograms of 1,000 Ω estimates given 1,000 datasets simulated under either of two constrained histories. The simulated histories in panels A and C involve simultaneous divergence across ten population pairs (Ω = 0.0; all *τ *= 1.8), whereas panels B and D are from histories involving two different divergence events across the 10 population pairs (Ω = 0.1; two splitting at *τ *= 1.0 and eight splitting at *τ *= 2.0). Panels A and B are using small sample sizes (≤ 5 individuals per population pair), whereas panels C and D are using samples of 10 individuals per population pair. The actual sample sizes used for panels A and B are species pair 1: 1, 2; pair 2: 3, 2; pair 3: 1, 1; pair 4: 2, 2; pair 5: 2, 3; pair 6: 2, 1; pair 7: 1, 1; pair 8: 1, 3; pair 9: 3, 1; pair 10: 2, 1.

The simulation analysis demonstrated that msBayes can usually distinguish simultaneous divergence from temporal incongruence in divergence, even with sparse sampling of individuals. The estimates of Ω were not markedly improved by sampling 20 individuals per population pair (10 each population) when compared to sampling 2–5 individuals per population pair (1–3 each population; Figure. 3). However, Ω is being overestimated under both sample sizes and this upward bias is stronger with larger sample sizes when true Ω = 1. Therefore, simultaneous divergence is easier to correctly reject with larger sample sizes. Root mean square error (RMSE) for estimating Ω was < 0.12 when the true history was simultaneous divergence (Ω = 0), and RMSE was < 0.18 when the true history involved 2 different divergence events across 10 population pairs (Ω = 0.1). It is encouraging that one can obtain fair estimates with so few samples per population pair and that two samples per population pair can be analyzed by msBayes.

An attractive benefit of an ABC method such as msBayes is that one can perform this estimator evaluation relatively quickly. Simulating data from parameters drawn from the prior is only done once per set of conditions (sample size/history) and can be done in approximately 5 hours per population pair on a 2 GHz linux computer. The computational time can be further reduced as the simulations can be run parallel on multiple processors. Because the acceptance/rejection step is decoupled from simulating the prior, multiple estimates from a series of simulated datasets can be accomplished without re-simulating the prior each time. The acceptance/rejection step for a single estimate is accomplished in one second to well under a minute such that 1,000 estimates can be obtained from 1,000 data sets simulated from fixed known parameter values in under an hour to within 24 hours on a single processor.

### General use and future development

The most important aspect of msBayes is that its flexible and modular nature will allow us and others to add in new features. This characteristic is essential for a phylogeographic software tool because phylogeographic studies are highly idiosyncratic. Using population genetic data to test how climate and/or geological changes result in biogeographic shifts, speciation, extinction and consequent changes in ecological interactions can involve a wide array of hypotheses and models that conform to no generality with regards to model complexity, parameterization and sampling. We therefore anticipate making several extensions to msBayes, and will encourage other bioinformaticians to make versions that suite particular difficult research questions. Furthermore, phylogeographic studies are most powerful when combined with independent evidence (or hypotheses) about past habitat distributions that are generated from other types of historic data and ecological distribution models [25]. Particular historical hypotheses can then be directly parameterized by paleo-distribution models and tested with genetic data within the msBayes framework using Bayes factors [26].

One feature we plan to include in future versions of msBayes is an option to simulate from the prior after constraining the number of divergence events per *Y *population pairs to one fixed number. This will then allow getting estimates for when these different isolation events took place as well as estimating which population pairs originated at either of the particular divergence events. Other upcoming features to be included are: 1.) multiple loci per population pair by expanding the summary statistic vector and adding additional hyper-parameters controlling mutation rate variation across loci; 2.) having more summary statistics available; 3.) allowing analysis of only one population pair at a time; 4.) testing multi-species colonization hypotheses; 5.) three or more population models (as opposed to two population models); 6.) allowing microsatellite data and 7.) automating the simulation testing procedure used to obtain estimator bias.

It should be noted that migration could hinder the ability of this method to correctly infer simultaneous divergence. Moderate migration in a subset of species/population pairs could cause the method to incorrectly support temporal discordance in divergence when the true history involved temporal congruence because migration can erase the genetic signal of isolation [27,28]. Although the Bayesian support for temporal concordance in divergence times would likely weaken if this happens in a subset of species/population pairs, we will explore using the summary statistic Var(*π*) as a means to tease apart migration from isolation as suggested in [29,30].

## Conclusion

The msBayes software pipeline will increasingly become an important tool as the field of comparative phylogeography progresses to become a more rigorous and statistical enterprise [5]. The program can obtain hyper-parameter estimates using hierarchical models in a reasonable amount of time without having the problems associated with convergence and mixing found in MCMC methods (Markov chain Monte Carlo). Because the estimation step is decoupled from the simulation step, one can quickly evaluate different ABC acceptance/rejection conditions and the choice of summary statistics. The method can reasonably distinguish biogeographic congruence from temporal incongruence, even with sparse sampling of individuals. Given the complex and idiosyncratic nature of testing multi-species biogeographic hypotheses, we envision msBayes as a powerful and flexible tool that is open for modification when faced with particularly difficult research questions. Finally, due to its flexible and modular design, msBayes will be a well-suited tool for the heterogeneous data sets that are emerging and being combined to test complex historical hypotheses.

## Availability and requirements

The installation instructions, documentation, source code and precompiled binary for msBayes are all available for download at  under an open source license (GNU Public License). The msBayes pipeline is comprised of several C and R programs that are run with a Perl "front-end" and runs on Linux, Mac OS-X, and most POSIX systems.

## List of Abbreviations used

ABC: Approximate Bayesian Computation

TSD: Test of simultaneous divergence

mtDNA: Mitochondrial DNA

## Authors' contributions

MJH developed the idea for using ABC within a hierarchical model to analyze multiple population pairs simultaneously. ES developed the finite sites version of D. Hudson's classic coalescent simulator (ms). MJH and NT developed C, R, and Perl routines and modified pre-existing R and C routines to comprise an ABC algorithm that makes use of a Hierarchical model. NT extensively developed the C and Perl routines that comprise the user version of msBayes now available. NT and MJH maintains the msBayes website and NT developed the installation configurations and precompiled binaries. All authors read and approved the final version of the manuscript.

## References

[B1] Avise JC (2000). Phylogeography: The history and formation of species.

[B2] Wen J (1999). Origin and the evolution of the eastern North American distinct distributions of flowering plants. Annu Rev Ecol Syst.

[B3] Lessios HA, Howard DJ, Berlocher S (1998). The first stage of speciation as seen in organisms separated by the Isthmus Panama. Endless Forms: species and speciation.

[B4] Barraclough TG, Nee S (2001). Phylogenetics and speciation. Trends Ecol Evol.

[B5] Knowles LL, Maddison WP (2002). Statistical phylogeography. Mol Ecol.

[B6] Edwards SV, Liu L, Pearl DK (2007). High-resolution species trees without concatination. Proc Natl Acad Sci USA.

[B7] Rannala B, Yang ZH (2003). Bayes Estimation of Species Divergence Times and Ancestral Population Sizes Using DNA Sequences From Multiple Loci. Genetics.

[B8] Hey J, Nielsen R (2007). Integration within the Felsenstein equation for improved Markov chain Monte Carlo methods in population genetics. Proc Natl Acad Sci USA.

[B9] Edwards SV, Beerli P (2000). Perspective: Gene divergence, population divergence, and the variance in coalescence time in phylogeographic studies. Evolution.

[B10] Hickerson MJ, Stahl E, Lessios HA (2006). Test for simultaneous divergence using approximate Bayesian computation. Evolution.

[B11] Thornton K, Andolfatto P (2005). Approximate Bayesian inference reveals evidence for a recent, severe bottleneck in a Netherlands population of Drosophila melanogaster. Genetics.

[B12] Excoffier L, Estoup A, Cornuet J-M (2005). Bayesian analysis of an admixture model with mutations and arbitrarily linked markers. Genetics.

[B13] Estoup A, Beaumont BA, Sennedot F, Moritz C, Cornuet J-M (2004). Genetic analysis of complex demographic scenarios: spatially expanding populations of the cane toad, *Bufo marinus*. Evolution.

[B14] Tallmon DA, Luikart G, Beaumont BA (2004). Comparative evaluation of a new effective population size estimator based on approximate Bayesian computation. Genetics.

[B15] Beaumont MA, Zhang W, Balding DJ (2002). Approximate Bayesian computation in population genetics. Genetics.

[B16] Weiss G, von Haeseler A (1998). Inference of population history using a likelihood approach. Genetics.

[B17] Watterson GA (1975). On the number of segregating sites in genetic models without recombination. Theor Popul Biol.

[B18] Nei M, Li W (1979). Mathematical model for studying variation in terms of restriction endonucleases. Proc Natl Acad Sci USA.

[B19] Hickerson MJ, Dolman G, Moritz C (2006). Comparative phylogeographic summary statistics for testing simultaneous vicariance across taxon-pairs. Mol Ecol.

[B20] Hudson RR (2002). Generating samples under a Wright-Fisher neutral model of genetic variation. Bioinformatics.

[B21] Hey J, Nielsen R (2004). Multilocus methods for estimating population sizes, migration rates and divergence time, with applications to the divergence of *Drosophila pseudoobscura *and *D. persimilis*. Genetics.

[B22] Beaumont MA, Rannala B (2004). The Bayesian revolution in genetics. Nat Rev Genet.

[B23] James W, Stein C (1960). Estimation with quadradic loss. Proceedings of the Fourth Berkeley Symposium on Mathematical Statistics and Probability: 1960.

[B24] Gelman A, Carlin JB, Stern HS, Rubin DB (1995). Bayesian Data Analysis.

[B25] Hugall A, Moritz C, Moussalli A, Stanisic J (2002). Reconciling paleodistribution models and comparative phylogeography in the Wet Tropics rainforest land snail *Gnarosophia bellendenkerensis *(Brazier 1875). Proc Natl Acad Sci USA.

[B26] Kass RE, Raftery A (1995). Bayesian factors. Journal of the American Statistical Association.

[B27] Kalinowski ST (2002). Evolutionary and statistical properties of three genetic distances. Mol Ecol.

[B28] Slatkin M (1985). Gene flow in natural populations. Ann Rev Ecol Syst.

[B29] Wakeley J (1996). The variance of pairwise nucleotide differences in two populations with migration. Theor Popul Biol.

[B30] Wakeley J (1996). Distinguishing migration from isolation using the variance of pairwise differences. Theor Popul Biol.

